# Resorbable Suture Is a Suitable Surgical Repair Material in a Rat Achilles Rupture and Repair Model

**DOI:** 10.2106/JBJS.OA.26.00079

**Published:** 2026-05-12

**Authors:** Natalie K. Gilmore, Sophie V. Orr, Selena K. Lam, Blaine A. Christiansen, Keith Baar

**Affiliations:** 1 Molecular, Cellular, and Integrative Physiology Graduate Group, University of California, Davis, California; 2 Department of Orthopedic Surgery, University of California, Davis, California; 3 Department of Neurobiology, Physiology, and Behavior, University of California, Davis, California; 4 Department of Physiology and Membrane Biology, University of California, Davis, California

## Abstract

**Background:**

Treatments for Achilles tendon rupture (ATR) are insufficient because they often do not result in full recovery and return to activity. There is no universal standard protocol for ATR, with surgeons using different repair techniques and materials. Some surgeons prefer nonresorbable (NR) suture due to the superior tensile strength, and others use resorbable (RES) because they decrease foreign body reactions.

**Methods:**

To establish a model for future interventional studies, we performed ATRs on 3-month-old female Sprague-Dawley rats using either NR or RES suture to repair the tendon and collected tissues at 1 and 2 months.

**Results:**

No significant differences in mechanical or material properties as a function of suture type were noted. Independent of suture type, tendons were wider at 1 month and both wider and thicker at 2 months, resulting in large increases in cross-sectional area (CSA) at each time point. The repaired tendons were stronger than the uninjured contralateral tendons at 1 month, but their strength decreased from the first month to the second despite increasing in CSA during this time, indicating progressive deterioration of tissue quality.

**Conclusions:**

Our data suggest that without loads in excess of body weight, the healing process becomes maladaptive after repair regardless of suture material.

**Level of Evidence/Clinical Relevance:**

These experiments provide Level I pre-clinical evidence showing that RES suture performs at least as well as NR in all physiological measures following repair, indicating it is the preferable repair material for ATR. See Instructions for Authors for a complete description of levels of evidence.

## Introduction

Tendons are dense connective tissues that link muscle to bone and absorb and transfer force from muscle contraction through the skeleton to facilitate movement. Healthy tendon is composed primarily of collagen, proteoglycans, water, enzymes, growth factors, and cytokines with tenocytes (tendon-specific fibroblasts) embedded throughout an aligned extracellular matrix (ECM) between collagen bundles^1^. Tendon injuries are common, accounting for half of all musculoskeletal injuries^2^ and are responsible for enormous economic and personal costs: Approximately $265 billion was spent directly on musculoskeletal injuries in the United States in 2016^3^.

The Achilles tendon is the largest and strongest in the body, connecting the soleus and gastrocnemius muscles of the posterior compartment of the lower leg to the calcaneus. The incidence of Achilles tendon rupture (∼31 ruptures per 100,000 people) continues to increase year on year and affects men at a 3:1 ratio compared with women^4-6^. Whether to perform surgery, which surgery to use, and the timing of rehabilitation protocols including whether and how long to immobilize the ankle following rupture remain open questions^7-9^. Surgical repair is associated with a lower risk of Achilles tendon rerupture, and better ultimate performance including higher return to activity rates^10^, but also has a higher risk for complications including wound problems^8^. Tendon lengthening is a key clinical measure following Achilles tendon repair (ATR) because it commonly occurs, causes biomechanical impairments^11^, and is negatively correlated with clinical outcomes. Therefore, avoiding tendon lengthening is a primary goal of treatment and rehabilitation^12,13^. Surgical repair reduces tendon lengthening and scar formation^8^. Therefore, the surgical option is generally recommended for healthy patients, but nonoperative treatment is similarly effective^10,14^.

There is no universal consensus regarding the optimal surgical ATR technique or material. A major dilemma is that while load on the native tendon is beneficial for regeneration and vital for preventing muscle and tendon atrophy, excess stress may have negative clinical consequences including rerupture, gap formation, and tendon lengthening^8^. Therefore, it is crucial to have a repair that is strong enough to resist gapping and rerupture during the critical healing phase before the formation of a strong fibrotic bond that can withstand load on its own. Repair strength can be modulated by factors including the repair technique (i.e., the types of knots used and how many knots are used to reinforce the tendon), or by the suture material, including properties such as thickness, whether the suture is a monofilament, looped, or braided, and whether it is resorbable (RES) or nonresorbable (NR).

American surgeons tend to prefer braided NR suture for tendon repair, while those in Europe tend to opt for strong monofilament suture or braided RES suture^8,15^. The benefits of NR suture include superior tensile strength, higher knot security, and holding resistance. Surgeons may also choose to use NR suture from fear that RES suture loses strength too early during the critical phases of tendon healing. A potential downside of NR suture, aside from the risk of foreign body reactions^16,17^, is that the suture is stiffer than even healthy tendon and may therefore stress-shield the native tissue long into the rehabilitation process preventing the native tissue from getting the load stimulus it needs to regenerate optimally^15^. This process is well described in bone with metal implants^18^. By contrast, as RES suture degrades, it should allow the native tissue to progressively take on more of the load. Over time, this may strengthen the native tissue better than when NR suture is used. The current study was designed to determine the role of suture material in tendon regeneration in a rat model. We hypothesized that using a resorbable suture to perform the repair would minimize stress-shielding and improve tendon healing leading to a stronger tendon without lengthening.

## Results

### Morphometry

**Fig. 1:**
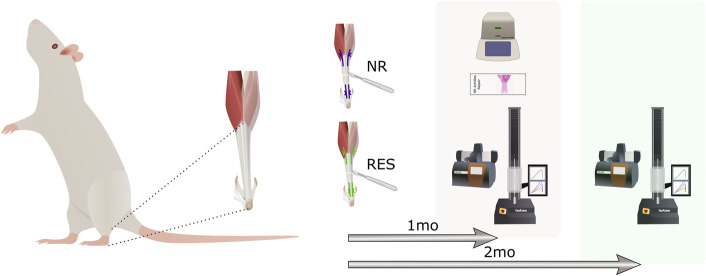
Schematic of experimental design. Achilles tendons were transected and repaired using either RES or NR sutures before collection at 1 month for tendon mechanics, collagen, and thermal properties (n = 8 rats per suture type; unilateral repair), histology, and PCR (n = 6 rats per suture type; bilateral repair), or 2 months for mechanics and collagen (n = 8 per suture type; unilateral repair). Total animals used in the study was n = 38. NR = nonresorbable, and RES = resorbable.

**Fig. 2:**
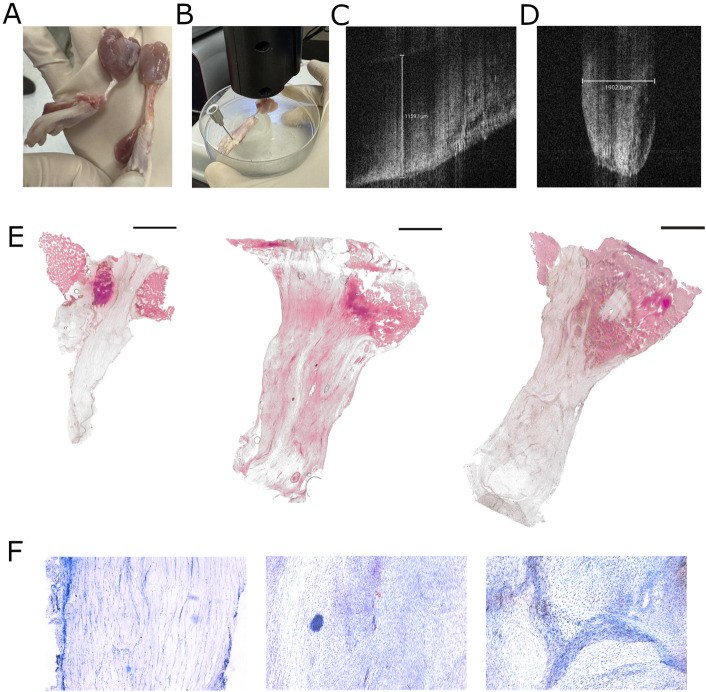
Achilles tendon morphology. **Fig. 2-A** Gross appearance of healthy (left) and contralateral injured (right) Achilles muscle-tendon-bone units 1 month after surgical repair. **Fig. 2-B** Preparation of Achilles tendon for OCT measurements of tendon width and thickness. **Fig. 2-C** Width (proximal to distal) and **Fig. 2-D** thickness (anterior to posterior) are determined using the Lumedica LabScope program. **Fig. 2-E** Representative intact longitudinal H&E-stained control (left) and Achilles tendons repaired with RES (center) and NR (right) suture (20×, scale bar = 2000 µm). **Fig. 2-F** Zoomed image of the tendons above to show cell number and collagen alignment for CON (left), RES (center), and NR (right). NR = nonresorbable, and RES = resorbable.

**Fig. 3:**
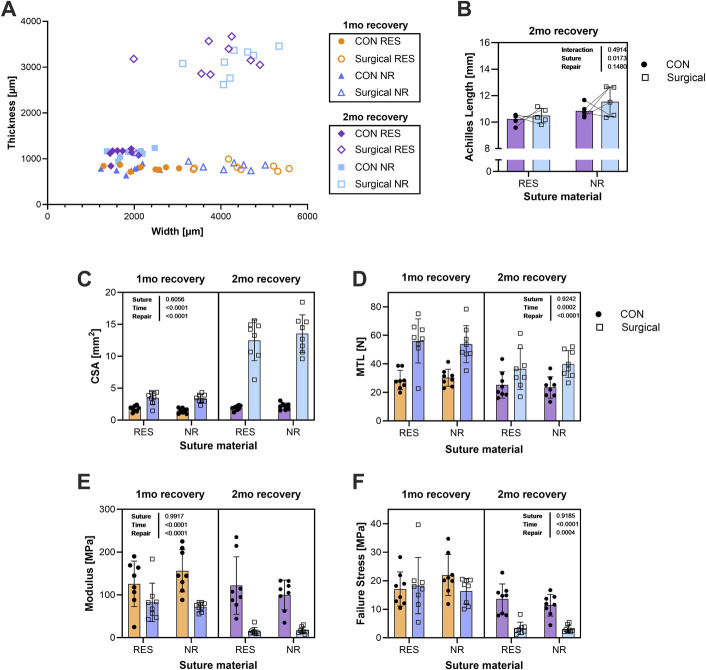
Achilles tendon morphometry and mechanical properties. **Fig. 3-A** Achilles tendon thickness and width were plotted on an *x*-*y* axis, allowing visualization of 2-dimensional changes in the size of the repaired tendon from 1-month (phase of increased width) to 2-months (phase where thickness increased) recovery. **Fig. 3-B** Tendon physiological length measured at 2 months indicates that there was statistically significant tendon lengthening in the NR group but no tendon lengthening in the RES suture group. Repair suture material (NR vs. RES) did not affect the following mechanical or material properties: cross-sectional area (CSA; **Fig. 3-C**), maximal tensile load (MTL; **Fig. 3-D**), modulus (**Fig. 3-E**), or failure stress (**Fig. 3-F**), but “repair” (versus the uninjured contralateral leg) and “time” (1-month vs. 2-month recovery) were significantly different (**Figs. 3-C through 3-F**). P-values are reported for 2-way ANOVA (n = 5 per group; (**Fig. 3-B**) and 3-way ANOVA (n = 8 per group; **Figs. 3-C through 3-F**), highlighting changes of the tendon scar healing process over these 2 time points. ANOVA = analysis of variance, CSA = cross-sectional area, MTL = maximal tensile load, NR = nonresorbable, and RES = resorbable.

We performed Achilles tendon transection and repair surgeries using either RES or NR suture and measured histological, mechanical, and matrix changes within the scar (Fig. 1). H&E staining (Fig. 2-F) comparing the longitudinal structure of representative control (CON) tendons with tendons 1 month after repair with RES and NR suture. Compared with CON, both repaired tendons were thicker and had a disorganized matrix with less parallel collagen (Fig. 2-F; left = healthy (CON), right = 1-month postrepair). Using optical coherence tomography (OCT) imaging (Fig. 2-B), we determined the thickness (Fig. 2-C) and width (Fig. 2-D) of the tendons and plotted the data on an X-Y plot (Fig. 3-A). The Achilles initially increased in width before growing thicker at 2 months. Tendon length was not different between CON and RES but was significantly longer with NR repaired tendons vs. CON at 2 months, indicating that repair with RES suture prevented tendon lengthening, while NR did not. Achilles CSA was not different as a function of suture type, but it was higher in the repaired tendons vs. CON, and dramatically higher at 2 months versus 1 month recovery (Fig. 3-C).

### Mechanics

Mechanical testing of the Achilles tendons showed that suture type did not influence maximal tensile load (MTL), modulus (normalized stiffness), or failure stress (Figs. 3-D, 3-E, and 3-F). However, we observed differences between healing and healthy tendons. The MTL was higher in the repaired legs vs. CON, and MTL at 1-month recovery was higher than at 2 months despite being significantly larger at 2 months, indicating that the tissue quality worsened later in the remodeling phase (Fig. 3-C). Repaired tendons had lower modulus than CON at both timepoints and far lower modulus at 2 months than at 1 month (Fig. 3-E). Failure stress was also worse in the repaired tendon at 2 months (Fig. 3-F). As modulus and failure stress are measures that are normalized by area, the large decreases in these 2 measures in our repaired tendons from 1 to 2 months are driven by the increase in CSA. Our analysis of TA tendons confirmed there were no significant differences in the biomechanics of the ipsilateral and contralateral legs (Fig. S1).

### Collagen Content

**Fig. 4:**
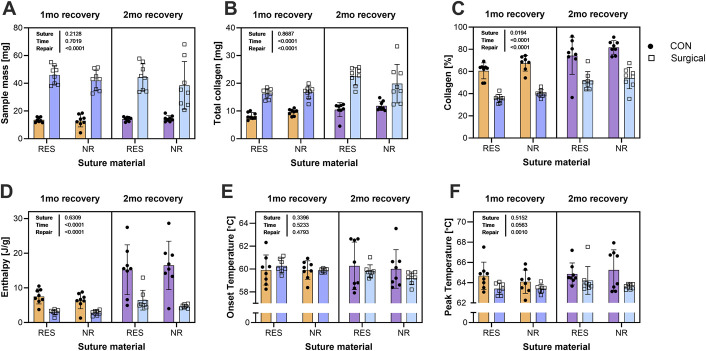
Achilles tendon collagen content and thermal properties. Postmechanically tested Achilles tendons were processed for the determination of collagen content (**Figs. 4-A, 4-B, and 4-C**) and thermodynamic properties (4 mm biopsy punch of central core; **Figs. 4-D and 4-E**) and analyzed by 3-way ANOVA. **Fig. 4-A** Tendon dry mass, (**Fig. 4-B**) total collagen content, and (**Fig. 4-C**) collagen content normalized to total dry mass. Suture type produced no statistically significant differences (**Figs. 4-A through 4-F**), but repaired tendons had altered collagen content (**Figs. 4-A, 4-B, and 4-C**) as well as enthalpy (**Fig. 4-D**) and peak temperature (**Fig. 4-F**).

The dry mass (Fig. 4-A) was similar in the tendons at 1 and 2 months, indicating that the increase in CSA between 1 and 2 months could largely be accounted for by a decrease in tissue density. The overall collagen content (Fig. 4-B) was not different with suture type but increased in repaired tendons vs. CON and from 1 to 2 months, indicating that collagen continued to be deposited after the first month. The collagen content was significantly higher for all comparisons, with a higher percentage in the NR group vs. the RES group and at 2 months vs. 1 month.

### Thermal Properties

Enthalpy was higher in CON than the repaired legs, and higher at 2 months vs. 1 month (Fig. 4-D). There were no differences in the temperature at the onset of denaturation with suture material, time, or repair (Fig. 4-E). While suture type had no effect on peak temperature, this measure was lower for repaired tendons vs. CON and trended toward being significantly higher (p = 0.0563) at 2 months vs. 1 month.

### Gene Expression

**Fig. 5:**
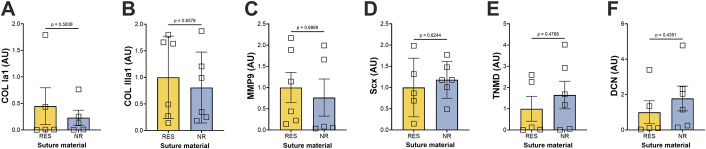
qPCR analysis of tendon genes comparing RES and NR suture vs. uninjured control tendon 1 month after repair. Gene expression levels of NR and RES Achilles tendon scars in the 1-month recovery group (n = 6 per group) were normalized to the housekeeping gene glyceraldehyde-3-phosphate dehydrogenase (GAPDH). Data are presented as relative fold change compared with an uninjured control Achilles tendon. P-values were calculated using the Welch corrected *t*-test. NR = nonresorbable, and qPCR = Quantitative PCR

There were no differences in gene expression as a function of suture type on the selected tendon genes measured (Figs. 5-A through 5-F). There was high variability in all genes across suture types, likely because RNA was isolated from shavings of the whole tendon and not just the scar region. The result was that no significant differences in gene expression were observed.

### Bone Microstructure

Neither surgical repair nor suture type affected calcaneus bone morphology 1 month after repair (Supplemental Figs. S2–S4). μCT showed no significant differences as a function of repair or suture material (p > 0.05 for all measures).

## Discussion

In our acute Achilles tendon transection and repair model, there were no postsurgical ruptures despite immediate loading. Furthermore, resorbable sutures provided suitable repair strength to prevent tendon lengthening. While we found a higher percentage of collagen in tendons repaired with NR suture (Fig. 4-C), the tendons repaired with RES suture had similar mechanical and material properties relative to those repaired with NR suture (Fig. 3), indicating the additional collagen in the NR group likely is indicative of greater scar material that did not contribute to tissue function. Another key finding was that, at 2 months, tendons repaired with RES suture were no different in length than the contralateral control tendon, whereas tendons repaired with NR suture were longer on average, indicating that RES suture prevented lengthening as well or better than NR. The fact that there was no change in TA tendon mechanics (Fig. S1) or calcaneal bone microstructure (Figs. S2–S4) indicates that the animals were loading the limb normally. This suggests that normal physical activity is not sufficient to improve Achilles mechanics after repair regardless of the suture type used.

Previous studies have evaluated RES and NR suture for Achilles repair in both human and animal models, with results indicating RES suture is a suitable repair material. O’Broin et al. compared the outcomes of PDS (RES) and prolene (NR) sutures in rabbit flexor digitorum longus tendon repairs up to 1 month^19^. They found no difference in tensile strength of the repairs but reduced foreign body reactions in the RES group. In humans, Kocaoglu et al. randomized 48 patients to receive either RES or NR suture (No. 2 braided nonabsorbable polyethylene terephthalate suture vs. No. 2 braided absorbable polyglactin suture) for ATR and showed similar clinical outcomes as determined by American Orthopedic Foot & Ankle Society scores but reduced postoperative complications (0%) in the RES group compared with the NR group (12.5%)^20^. Park et al.^21^ randomized 40 patients to either braided absorbable polyglactin suture or braided nonabsorbable polyethylene terephthalate suture groups for ATR and found equal results up to 12-month follow-up. Similarly, a retrospective cohort study of 210 patients receiving resorbable or nonresorbable suture by Chen et al.^22^ suggested that functional and structural outcomes were better in the RES group even though the identical postoperative rehabilitation protocol was used. These improved outcomes included reduced tendon thickness, active gliding function, improved VISA-A scores, and reduced blood flow signals at 6 months. However, all outcome measures had equalized by 12 months. Like the current work, these studies concluded that RES suture was a viable material for tendon repair and avoids the problem of long-term foreign body reactions.

Both the rodent and human data suggest that RES sutures perform at least as well as NR sutures. The value of the current study is to provide a model, where postoperative interventions can be directly tested. For example, the model described here is well positioned to understand 3 key areas of interest that are hard to test in patients: (1) the role of load intensity and duration on the mechanical and structural properties of regenerating tendon, (2) how pharmaceutical interventions alter tendon regeneration, and (3) how nutritional or peptide interventions affect tendon healing.

### Repair on Achilles Structure and Function

Macroscopically, the healthy tendon appeared brilliant white with a fibroelastic texture, while injured tendon appeared gray-brown and amorphous as a result of disordered and haphazard healing with hypercellularity and vascular ingrowth^23,24^. Our surgically transected and repaired tendons were consistent with the literature^1,23^. The tendons were wider at the 1-month collection and then demonstrated increased thickness at the 2-month collection. The result was a progressive increase in CSA. The most likely explanation for the pattern of growth is that the sutures were placed in parallel across the width of the tendon. The initial response was likely to encase the sutures, making the tendon wider, before adding thickness to the scar. Though larger and stronger than healthy tendon, the repaired tendons had inferior material properties compared with the contralateral tendons and interestingly material properties worsened significantly from 1 to 2 months.

### Changes in the Matrix of the Achilles Tendon

One interesting finding was that in both groups, the control Achilles tendons increased in collagen concentration from 1 to 2 months of repair (Fig. 4). Concomitant with the increase in the percent collagen within the control tissue there was an increase in enthalpy, suggesting that there was an increase in the packing density of the collagen fibrils. A similar increase in collagen and packing density is seen in the scar between 1 and 2 months. The onset temperature, which indicates young, dynamic, and easily broken cross-links, was unchanged, whereas the peak melting temperature, which indicates mature stable cross-links, was lower in the repaired leg. These data are consistent with an increase in turnover at the core of the repaired Achilles and an effort to increase collagen turnover in both the control and repaired tendon. Initially, we thought the increase in collagen and enthalpy in the control Achilles could be the result of favoring the healthy leg; however, neither the bone nor the tibialis anterior tendon data suggest significant changes in loading. Therefore, the reason for the increased collagen in the control Achilles needs to be investigated further.

### Limitations

Animal models are different than humans, and outcomes may differ. Our data add to a pool of data on tendon repair in animal models—we would point interested readers to the 2020 review by Notermans et al.^25^ that aggregates the main existing data from animal studies of tendon repair. These studies have primarily been conducted in rats, in both sexes, using different repair techniques, and postoperative loading conditions. Rats are quadrupeds and distribute load differently than humans on an injured leg. We modeled rupture by transecting healthy tendons, whereas in humans, Achilles ruptures are always predated by degeneration^23^ resulting in frayed ends for the repair. The age of the animals is also a potential limitation, as 3-month-old rats are comparatively younger than the typical age when humans rupture their Achilles tendons—rupture classically occurs in middle-aged individuals, in whom the vascular impairment and degenerative changes are more prominent^26^. However, we chose not to use an older animal model since we were most interested in modeling optimal regeneration of the Achilles, and this occurs most frequently in the young. Even though this is a smaller population, they put greater demands on the repair than the elderly. In the rat, this is reflected as greater home cage ambulation. Therefore, if there were to be a weakness due to completing the repair with RES sutures, this would be more evident in young/active individuals.

Female rat tendons heal differently and have a higher CSA that more closely mimics what we see in humans. For this study, we also decided to include only female rats because unlike females who maintain a weight of approximately 300 grams, male Sprague-Dawley rats continue to grow throughout adulthood, become less active due to an impaired ability to ambulate within the cage, and develop insulin resistance, as we report in a recent study (Hayden et al.)^27^. In fact, male Sprague-Dawley rats have been used to create the UC Davis type 2 diabetes mellitus rat model^28^. Since insulin resistance has been shown to impair tendon function^29,30^, we felt that including male animals would increase confounding issues and make conclusions more difficult to draw.

There are other methodological limitations to this study, including that it was not possible to blind the research team to the identity of the rats during surgery and tendon tensile testing because the NR and RES sutures were different colors. However, *ex vivo* assays were blinded wherever possible. Another caveat is that we performed DSC on the Achilles tendons after they had been mechanically tested to minimize the number of experimental animals used. Loading to failure may cause damage to the tissue that affects the thermal properties measured. However, since all tendons were tested using the same protocol, the damage should be consistent between specimens, and the data were internally consistent. Since our primary experimental outcome measure was mechanics, only a small subset of tendons were used for histology. Therefore, quantitative histological scoring was not possible. Finally, future work using this model should perform gait analysis, which would be useful to determine any functional biomechanical differences between groups.

### Future Directions

Without loads greater than body weight, the mechanics of the regenerating Achilles decreased. Whether incorporating a loading component into postsurgical recovery period can improve the mechanics of the healing tendon still needs testing. If there is a negative effect of RES sutures on repair strength, these differences may only become apparent when loads in excess of body weight are placed on the tissue. This could be accomplished by adding an exercise protocol such as treadmill running or stimulating the gastrocnemius muscle to load the tendon during the recovery period.

## Conclusions

Our data suggest that in a healthy female rat ATR model, RES suture performed at least as well as NR suture in all physiological measures. RES suture also prevented tendon lengthening and provided the initial strength necessary for the repair. The increase in CSA and decreased Achilles tendon material properties at 2 months indicate that from 1 to 2 months post-ATR, the healing process becomes maladaptive, even with immediate postoperative weight bearing and voluntary cage activity.

## Funding

The study was funded in part by the Peretz Family Fund for Connective Tissue Research.

N.K. Gilmore was supported by the UC Davis T32 in Pharmacology (T32 GM144303).

## Consent for Publication

All authors consented to publication of the data.

## Availability of Data and Material

Raw data are available upon reasonable request.

## Authors' Contributions

K. Baar and N.K. Gilmore conceived the study. N.K. Gilmore, S.V. Orr, and S.K. Lam collected and analyzed the data. N.K. Gilmore and K. Baar wrote the manuscript. S.V. Orr and B.A. Christiansen edited the manuscript.

## Appendix

Supporting material provided by the authors is posted with the online version of this article as a data supplement at jbjs.org (http://links.lww.com/JBJSOA/B188). This content was not copyedited or verified by JBJS.
